# Outcomes of rheumatic fever in Uganda: a prospective cohort study

**DOI:** 10.1016/S2214-109X(23)00567-3

**Published:** 2024-02-14

**Authors:** Scott H Wirth, Jafesi Pulle, JangDong Seo, Nicholas J Ollberding, Doreen Nakagaayi, Craig Sable, Asha C Bowen, Tom Parks, Jonathan Carapetis, Emmy Okello, Andrea Beaton, Emma Ndagire

**Affiliations:** a Heart Institute, Cincinnati Children's Hospital Medical Center, Cincinnati, OH, USA; b Uganda Heart Institute, Kampala, Uganda; c Children's National Medical Center, Washington, DC, USA; d Telethon Kids Institute, University of Western Australia and Perth Children's Hospital, Nedlands, WA, Australia; e Imperial College London, London, UK; f University of Cincinnati College of Medicine, Cincinnati, OH, USA; g Division of Biostatistics and Epidemiology, Department of Pediatrics, Cincinnati Children's Hospital Medical Center, University of Cincinnati, Cincinnati, OH, USA

## Abstract

**Background:**

Rheumatic heart disease is the largest contributor to cardiac-related mortality in children worldwide. Outcomes in endemic settings after its antecedent illness, acute rheumatic fever, are not well understood. We aimed to describe 3–5 year mortality, acute rheumatic fever recurrence, changes in carditis, and correlates of mortality after acute rheumatic fever.

**Methods:**

We conducted a prospective cohort study of Ugandan patients aged 4–23 years who were diagnosed with definite acute rheumatic fever using the modified 2015 Jones criteria from July 1, 2017, to March 31, 2020, enrolled at three rheumatic heart disease registry sites in Uganda (in Mbarara, Mulago, or Lira), and followed up for at least 1 year after diagnosis. Patients with congenital heart disease were excluded. Patients underwent annual review, most recently in August, 2022. We calculated rates of mortality and acute rheumatic fever recurrence, tabulated changes in carditis, performed Kaplan-Meier survival analyses, and used Cox regression models to identify correlates of mortality.

**Findings:**

Data were collected between Sept 1 and Sept 30, 2022. Of 182 patients diagnosed with definite acute rheumatic fever, 156 patients were included in the analysis. Of these 156 patients (77 [49%] male and 79 (51%) female; data on ethnicity not collected), 25 (16%) died, 21 (13%) had a cardiac-related death, and 17 (11%) had recurrent acute rheumatic fever over a median of 4·3 (IQR 3·0–4·8) years. 16 (24%) of the 25 deaths occurred within 1 year. Among 131 (84%) of 156 survivors, one had carditis progression by echo. Moderate-to-severe carditis (hazard ratio 12·7 [95% CI 3·9–40·9]) and prolonged PR interval (hazard ratio 4·4 [95% CI 1·7–11·2]) at acute rheumatic fever diagnosis were associated with increased cardiac-related mortality.

**Interpretation:**

These are the first contemporary data from sub-Saharan Africa on medium-term acute rheumatic fever outcomes. Mortality rates exceeded those reported elsewhere. Most decedents already had chronic carditis at initial acute rheumatic fever diagnosis, suggesting previous undiagnosed episodes that had already compounded into rheumatic heart disease. Our data highlight the large burden of undetected acute rheumatic fever in these settings and the need for improved awareness of and diagnostics for acute rheumatic fever to allow earlier detection.

**Funding:**

Strauss Award at Cincinnati Children's Hospital, American Heart Association, and Wellcome Trust.

## Introduction

Rheumatic heart disease is an acquired chronic valvular disease that is the cumulative outcome of severe or recurrent episodes of acute rheumatic fever, an acute systemic inflammatory condition that results from pharyngeal or skin and soft tissue infections with group A *Streptococcus pneumonia*.^1^ Through prevention efforts, rheumatic heart disease has largely been eliminated from high-income countries, but the disease remains the number one cause of cardiovascular-based morbidity and mortality and disability-adjusted life-years in young people worldwide, the burden of which is overwhelmingly borne in resource-limited settings such as sub-Saharan Africa. Over 40 million people have rheumatic heart disease, which accounts for over 300 000 deaths annually.^2^ Secondary antibiotic prophylaxis with intramuscular benzathine penicillin G is the only intervention known to reduce progression and poor outcomes along the continuum from acute rheumatic fever to rheumatic heart disease, although the uptake of benzathine penicillin G remains suboptimal in areas where acute rheumatic fever and rheumatic heart disease are most endemic.^3^

The past decade has seen a relative increase in research around rheumatic heart disease, but there has not been a parallel increase in research regarding its antecedent illness acute rheumatic fever. In part, this is because acute rheumatic fever is difficult to diagnose and characterise in resource-limited settings.^4^, ^5^ Although acute rheumatic fever rarely leads to mortality in high-income countries, there is a substantial knowledge gap in our understanding of the clinical presentation, burden, and outcomes related strictly to acute rheumatic fever in resource-limited settings. Importantly, this deficit also hinders any improvements in the diagnosis, management, and outcomes of patients with acute rheumatic fever, particularly regarding prevention and therapeutics.

**Research in context**

**Evidence before this study**

We searched PubMed with the terms “acute rheumatic fever” OR “rheumatic heart disease” AND “outcomes” OR “predictors” OR “mortality” OR “recurrence” for papers in English and French published between Jan 1, 1995, and Aug 9, 2023. There were no contemporary studies from sub-Saharan Africa reporting outcomes after acute rheumatic fever. Existing studies on outcomes primarily concentrated on retrospective cohorts from South America, southeast Asia, and Oceania that used tertiary patient data collected from hospital or government mortality records and were unable to distinguish outcomes related to acute rheumatic fever from those related to rheumatic heart disease.

**Added value of this study**

We assessed the rates of adverse outcomes, progression of carditis, and correlates of mortality in a community-based cohort of Ugandan children and young adults (aged 4–23 years) who were first diagnosed with acute rheumatic fever between July 1, 2017, and March 31, 2020. To our knowledge, this is the first contemporary study investigating medium-term outcomes after acute rheumatic fever in the highly endemic region of sub-Saharan Africa. We found high rates of mortality, which were associated with the presence of advanced carditis at the time of first diagnosis. In those who survived, however, cardiac involvement improved over time.

**Implications of all the available evidence**

We strongly suspect the high rates of mortality in our study to be due to late presentation to care after previously undetected acute rheumatic fever episodes. These findings underscore the low rate of acute rheumatic fever recognition and the associated opportunity loss with regard to morbidity and mortality in resource-limited settings such as sub-Saharan Africa. Changing this paradigm will require improved case detection. Our findings suggest that increased access to echocardiography might be helpful, but a simplified, field-deployable, point-of-care test or biomarker assay for detecting group A *Streptococcus* and acute rheumatic fever could be more efficient and effective in achieving this end.

In 2018, WHO recognised that mitigating this knowledge gap might decrease the global burden of rheumatic heart disease and resolved to improve our understanding of the epidemiology of acute rheumatic fever.^6^ In response, from July 1, 2017, to March 31, 2020, we conducted an epidemiologic study in Uganda that used active case-finding strategies to determine the community incidence of acute rheumatic fever.^4^, ^5^ Uganda is a country in eastern Africa and is home to approximately 44 million people, more than half of whom are younger than 18 years. Our previous study found an incidence of 13–25 per 100 000 person-years within this population, or about 6000–11 000 annual incident cases, establishing that acute rheumatic fever was present in communities at an endemic rate despite diagnostic rates outside the context of research remaining exceptionally low.^4^, ^5^

In the current study, we aimed to build on our incidence study findings by describing post-acute rheumatic fever outcomes in children and young adults. Specifically, we aimed to determine rates and incidence of all-cause mortality, cardiac-related mortality, and acute rheumatic fever recurrence after diagnosis, to describe changes in carditis by echocardiography, and to identify correlates of cardiac mortality.

## Methods

### Study design and patients

We conducted a prospective cohort study of Ugandan patients aged 4–23 years who were diagnosed with definite acute rheumatic fever between July 1, 2017, and March 31, 2020. Additional inclusion criteria were enrolment in the Uganda national rheumatic heart disease registry (hereafter referred to as registry) at either the Mbarara, Mulago, or Lira sites, and completion of at least 1 year of post-diagnosis follow-up. Patients with congenital heart disease were excluded. Diagnosis was made according to the 2015 Jones criteria.^7^ Each participant had a unique observational period, starting at the diagnosis date and finishing at the date of death or most recent follow-up.

The registry was established in 2010 at the Uganda Heart Institute, Kampala, Uganda, the only tertiary cardiac centre in the country. In 2014, the registry expanded to urban regional hospitals in Mbarara (west) and Gulu (north), and in 2015 it began enrolling patients identified via outreach initiatives, including at the regional hospital in Lira (northeast) and the district hospital in Tororo (east). The registry employs a central staff of nurses, data managers, physician supervisors, and local nurses at research sites throughout the country, who together are responsible for following up patients, entering data, and performing quality checks.

This study was approved by the Uganda Heart Institute Research Ethics Committee (UHIREC0003) and designated as exempt by the Institutional Review Board of Cincinnati Children's Hospital Medical Center (#2022–0034). Written informed consent was obtained from all patients aged 18 years and older and from caregivers of all patients younger than 18 years, and written assent was obtained for all patients aged 8–17 years.

### Procedures

After acute rheumatic fever diagnosis, patients were enrolled in the registry and followed up per routine clinical practice. Patients were scheduled to receive secondary antibiotic prophylaxis injections by registry nurses every 28 days and annual review by registry clinicians, with all data entered into the registry. At each encounter, a review of symptoms was obtained and, if the symptoms raised concern for recurrent acute rheumatic fever, patients were evaluated via the modified Jones criteria. Echocardiograms were obtained 1 year after diagnosis and subsequently only as indicated clinically.

To supplement existing registry data, including imaging, and to capture as many patients as possible, we recruited patients to return for a dedicated study visit using an active recall process. Recruitment occurred from July 1 to July 31, 2022, during which patients or their caregivers were contacted by telephone, text message, or WhatsApp, invited to return, and informed of the visit's purpose and procedures, in their preferred language. Study visits occurred from Aug 1 to Aug 31, 2022. During these study visits, we obtained an updated verbal history to review interim acute rheumatic fever recurrences, hospitalisation admission, or surgeries, which we cross-referenced with registry records. Subsequently, we obtained an electrocardiogram and categorised the PR interval as normal or prolonged according to age-standardised normal reference values of 0·08–0·15 s for children aged 1–2 years, 0·09–0·16 s for children aged 3–7 years, 0·09–0·17 s for children aged 8–11 years, and 0·09–0·18 s for children older than 12 years and adults.^8^ We also obtained an echocardiogram, the protocol for which is described below. Finally, we reviewed hard-copy secondary antibiotic prophylaxis records to determine adherence, calculated as the number of received injections, divided by prescribed injections. For patients who could not return for the dedicated study visit, identical follow-up data were extracted from their most recent registry encounter. Separately, baseline data pertaining to the initial diagnosis were collected for each participant by extracting registry data on sex (self-reported as male or female at the time of initial enrolment), age, PR interval, echocardiogram findings, and modified Jones criteria.

Each participant who returned for the dedicated study visit in August, 2022, received a follow-up echocardiogram, performed by two research team members (SHW and EN) using a GE Vivid IQ ultrasound (General Electric, Chicago, IL, USA) or a Philips Lumify ultrasound (Philips, Amsterdam, Netherlands). Parasternal long axis and short axis view and apical four chamber and five chamber views were obtained with two-dimensional, colour, and spectral doppler echocardiography. Images were de-identified and exported to a cloud-based repository. Each follow-up echocardiogram was assigned a grade for mitral regurgitation, mitral stenosis, and aortic regurgitation by a team of experts (CS, EN, and DN), according to the expert consensus statement on evaluation of valvular heart disease.^9^ For patients who did not return to the dedicated study visit, the most recent echocardiogram in the registry was similarly encoded. Experts were masked to participant status and baseline echocardiogram findings. Two of the three experts had read echocardiograms at the time of initial diagnosis, minimising inter-reader variability between baseline and follow-up studies. Each baseline and follow-up echocardiogram was then assigned into one of three categories: no carditis, mild carditis, or moderate-to-severe carditis. No carditis was defined as no mitral regurgitation, no mitral stenosis, and no aortic regurgitation; mild carditis was defined as no more than mild mitral regurgitation and/or no more than mild aortic regurgitation and no mitral stenosis; moderate-to-severe carditis was defined as moderate-to-severe mitral regurgitation and/or moderate-to-severe aortic regurgitation and/or any mitral stenosis.^10^

All data were entered into a secure Research Electronic Data Capture (REDCap) database, hosted at Cincinnati Children's Hospital Medical Center, Cincinnati, OH, USA. Patients who met inclusion criteria but who died during the observation period were not present for the study visit and therefore follow-up data was not provided. Decedents were included in the primary and secondary analyses on mortality and risk factors but excluded from the secondary analysis on echocardiographic change and secondary antibiotic prophylaxis adherence.

### Outcomes

The primary outcome measures were mortality, cardiac-related mortality, and acute rheumatic fever recurrence. Mortality was reported by the caregiver in the re-recruitment contact call and cross-referenced with registry records, and was based off of verbal autopsy. To avoid the acute period, recurrence was defined as any episode occurring at least 3 months after the initial diagnosis that was diagnosed by a clinician and communicated to the patient or caregiver or documented in the registry. The secondary outcome measures were change in mitral regurgitation, mitral stenosis, aortic regurgitation, and carditis category between baseline and follow-up echocardiograms, and any covariate that imparted increased risk of all-cause mortality.

### Statistical analysis

Summary statistics were used to describe distribution (n, percentage) and central tendency (median, IQR) of baseline demographic and clinical variables and raw event rates and incidence rates were calculated for mortality, cardiac mortality, and acute rheumatic fever recurrence.

Follow-up time accrued from the date of acute rheumatic fever diagnosis until the date of death or censoring. Patients were censored on the date in which they were lost to follow-up or at the end of the follow-up period (Sept 1, 2022). Incidence rates were computed by dividing the number of new events over follow-up by the person-years at risk and multiplying them by 1000 to obtain rates per 1000 person-years of follow-up. All-cause mortality, cardiac-related mortality, and acute rheumatic fever recurrence were also evaluated using Kaplan-Meier analyses showing the probability of event-free survival over the study's observation period. Hazard ratios (HRs) and 95% CIs for correlates of mortality were identified using Cox proportional hazards regression models adjusted for age and sex. A p value of less than 0·05 was considered significant. Baseline and follow-up echocardiographic data were assessed using summary statistics. Statistical analysis was conducted using R version 4.2.1.

### Role of the funding source

The funders of the study had no role in study design, data collection, data analysis, data interpretation, or writing of the report.

## Results

**Figure 1:**
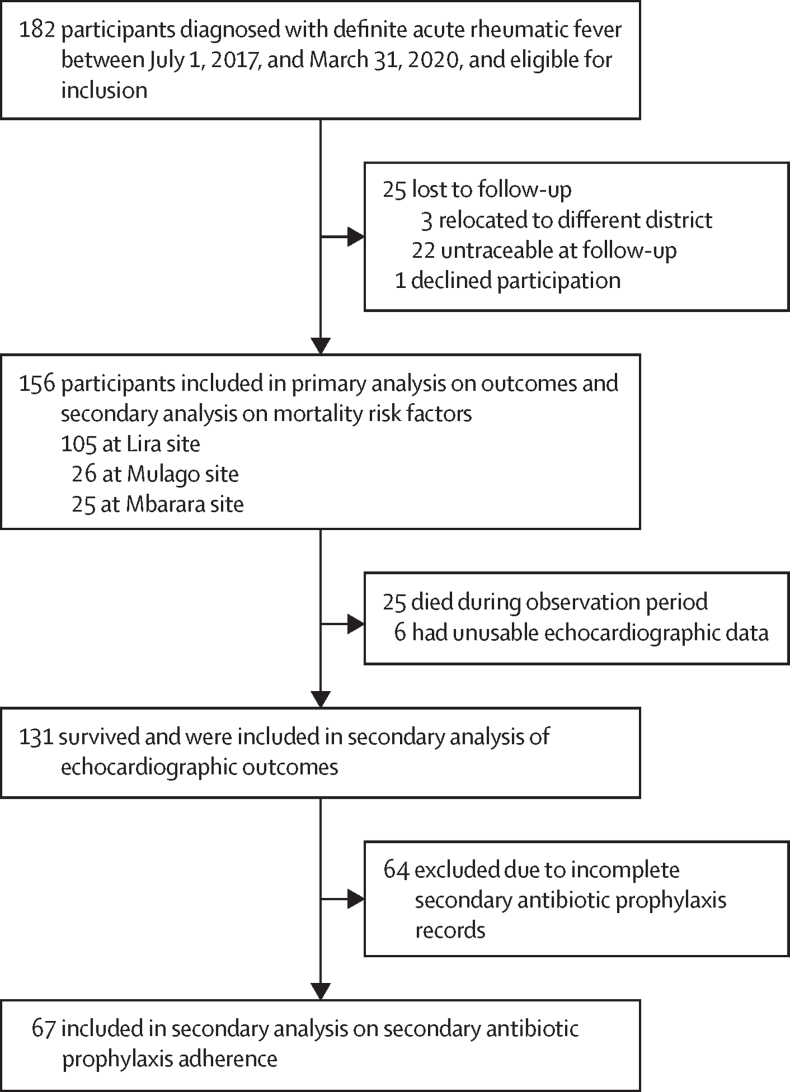
Study profile

Between July 1, 2017, and March 31, 2020, 182 patients were diagnosed with definite acute rheumatic fever and met the inclusion criteria. Of these patients, 113 (62%) returned for the dedicated study visit in August, 2022, 18 (10%) were unable to return for the study visit but had updated data available through their scheduled registry follow-ups, and 25 (14%) died; 25 (14%) patients were lost to follow-up due to relocation to other districts in Uganda or being untraceable due to outdated contact and one (<1%) patient declined study participation, and subsequently these patients were not included in the analyses (figure 1).

Of the 156 patients (77 [49%] male and 79 [51%] female) included in the primary analysis, 105 (67%) were enrolled at the Lira site, 26 (17%) at Mulago, and 25 (16%) at Mbarara. Median age was 9·5 (IQR 7·0–12·0) years at diagnosis and 13·5 (IQR 10·3–16·9) years at most recent follow-up, with a median observation period of 4·3 (IQR 3·0–4·8) years. Total follow-up was 574·2 person-years. 64 (41%) of 156 patients resided in permanent housing facilities and 113 (72%) of 156 patients attended day school. At the time of initial acute rheumatic fever diagnosis, the majority (109 [70%] patients) satisfied only one major Jones criterion, and 40 [37%] of the 109 patients satisfied two major Jones criteria. Additional information on the demographics, electrocardiogram and echocardiographic findings, and clinical history of the cohort are shown in table 1.

**Table 1:** Baseline demographic, electrocardiographic, echocardiographic, and clinical characteristics at initial acute rheumatic fever diagnosis

			**Lira site (n=105)**	**Mulago site (n=26)**	**Mbarara site (n=25)**	**Total (n=156)**
Demographics						
	Age, years		10 (7–13)	8·5 (6·1–10·9)	9 (7·5–10·5)	9·5 (7–12)
Sex (self-reported)						
	Male		50 (48%)	14 (54%)	13 (52%)	77 (49%)
	Female		55 (52%)	12 (46%)	12 (48%)	79 (51%)
Housing type						
	Permanent		39 (37%)	12 (46%)	13 (52%)	64 (41%)
	Semi-permanent		66 (63%)	14 (54%)	12 (48%)	92 (59%)
Number of individuals in household			6 (4·5–7·5)	6·5 (4·5–8·5)	6 (5–7)	6 (4·5–7·5)
Household members younger than 15 years			3 (2–4)	3 (1·6–4·4)	3 (2–4)	3 (2–4)
Attended school						
	No		21 (20%)	3 (12%)	0	24 (15%)
	Yes		84 (80%)	23 (88%)	25 (100%)	132 (85%)
School type						
	None		21 (20%)	3 (12%)	0	24 (15%)
	Day		72 (69%)	20 (77%)	21 (84%)	113 (72%)
	Boarding		12 (11%)	3 (12%)	4 (16%)	19 (12%)
Baseline electrocardiogram and echocardiogram findings						
	PR interval, ms		136 (123·8–148·3)	140 (122–158)	142 (125–159)	137 (124–150)
Mitral regurgitation						
	None		48 (45%)	13 (50%)	11 (44%)	72 (46%)
	Trivial		15 (14%)	1 (4%)	1 (4%)	17 (11%)
	Mild		9 (9%)	3 (11%)	3 (12%)	15 (10%)
	Moderate		9 (9%)	2 (8%)	3 (12%)	14 (9%)
	Severe		24 (23%)	7 (27%)	7 (28%)	38 (24%)
Mitral stenosis						
	None		102 (97%)	26 (100%)	25 (100%)	153 (98%)
	Mild		2 (2%)	0	0	2 (1%)
	Moderate		1 (1%)	0	0	1 (1%)
	Severe		0	0	0	0
Aortic regurgitation						
	None		70 (66%)	19 (72%)	17 (68%)	106 (67%)
	Trivial		6 (6%)	0	1 (4%)	7 (5%)
	Mild		16 (15%)	3 (12%)	3 (12%)	22 (14%)
	Moderate		9 (9%)	3 (12%)	2 (8%)	14 (9%)
	Severe		4 (4%)	1 (4%)	2 (8%)	7 (5%)
**Baseline Jones criteria**						
	Streptococcal evidence					
		Anti-streptolysin O titre	63 (60%)	20 (77%)	14 (56%)	97 (62%)
		Anti-DNAse B titre	76 (72%)	16 (62%)	21 (84%)	113 (72%)
		Throat culture	8 (8%)	0	0	8 (5%)
		Rapid antigen	38 (36%)	8 (31%)	14 (56%)	60 (39%)
	Quantity of major Jones criteria					
		One	67 (63%)	22 (84%)	21 (84%)	109 (70%)
		Two	34 (33%)	3 (12%)	3 (12%)	40 (26%)
		Three	4 (4%)	1 (4%)	1 (4%)	6 (4%)
	Major Jones criteria					
		Carditis	40 (38%)	4 (15%)	9 (36%)	53 (34%)
		Monoarthritis	9 (9%)	3 (12%)	0	12 (8%)
		Polyarthritis	41 (39%)	1 (4%)	6 (24%)	48 (31%)
		Polyarthralgia	49 (47%)	21 (81%)	12 (48%)	82 (53%)
		Subcutaneous nodules	1 (1%)	0	0	1 (1%)
		Erythema marginatum	0	0	0	0
		Chorea	6 (6%)	2 (8%)	3 (12%)	11 (7%)
	Quantity of minor Jones criteria					
		None	1 (1%)	1 (4%)	2 (8%)	4 (3%)
		One	12 (11%)	2 (8%)	3 (12%)	17 (11%)
		Two	53 (51%)	9 (35%)	13 (52%)	75 (48%)
		Three	38 (36%)	13 (49%)	7 (28%)	58 (37%)
		Four	1 (1%)	1 (4%)	0	2 (1%)
	Minor Jones criteria					
		Fever	103 (98%)	22 (85%)	21 (84%)	146 (94%)
		Monoarthralgia	1 (1%)	0	1 (4%)	2 (1%)
		Erythrocyte sedimentation rate ≥30 mm/h	67 (64%)	20 (77%)	7 (28%)	94 (60%)
		C-reactive protein ≥5 mg/L	62 (59%)	17 (65%)	18 (72%)	97 (62%)
		PR prolongation	3 (3%)	4 (15%)	3 (12%)	1 (6%)

Data are n (%) or median (IQR).

In the primary analysis, 25 (16%) of 156 patients died from any cause: 21 (13%) patients died from cardiac-related causes, three (one sepsis, one brain tumour, and one severe anaemia) died from non-cardiac causes, and one died from an unknown cause. Morbidity included 17 (11%) patients with acute rheumatic fever recurrence, 39 (25%) admitted to hospital for any reason, and six (4%) undergoing cardiac surgery. All surgeries comprised mechanical valve replacement. There were no interventional cardiac catheterisations. Incidence of all-cause mortality per 1000 person-years was 43·3 (95% CI 28·0–63·9), incidence of cardiac-related mortality per 1000 person-years was 36·4 (95% CI 22·5–55·6), and incidence of acute rheumatic fever recurrence per 1000 person-years was 29·4 (95% CI 17·2–47·1).

**Figure 2:**
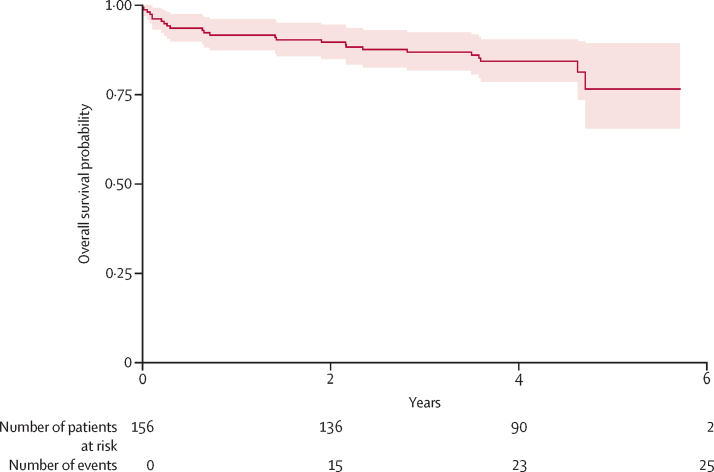
Kaplan-Meier survival curve showing freedom from all-cause mortality for Ugandan patients aged 4–23 years who were diagnosed with acute rheumatic fever from July 1, 2017, to March 31, 2020, and followed up by the Uganda rheumatic heart disease registry

In the Kaplan-Meier analysis, overall survival probability was 84% (95% CI 77·3–90·7). The last death occurred around 4 years post-acute rheumatic fever cardiac-related death. Most deaths occurred much earlier; 12 (48%) of 25 deaths occurred within 6 months, 16 (64%) within 1 year, and 20 (80%) within 3 years (figure 2). Survival probability for cardiac-related death was 86% (95% CI 82·2–89 8) with similar distribution of deaths (appendix 1 p 2). The Kaplan-Meier models show increasing right-censorship beginning at around 3·5 years. This finding was not entirely because of loss to follow-up, but also because each participant had a unique observational period.

Cox regression models adjusted for age and sex found that having a prolonged PR interval (HR 4·37 [95% CI 1·7–11·2]) or moderate-to-severe carditis (HR 12·7 [95% CI 3·96–40·9]) at the time of initial diagnosis was associated with increased all-cause mortality. Similarly, enrolment at the Mbarara site compared with the Lira site was associated with increased all-cause mortality (HR 3·1 [95% CI 1·1–8·75]). No significant associations were found for age, quantity of major Jones Criteria, or any singular Jones criterion beyond PR prolongation (table 2).

**Table 2:** Cox proportional hazards models adjusted for age and sex demonstrating hazard ratios for association between demographic and clinical covariates and cardiac mortality

		**Hazard ratio (95% CI)**	**p value**
Moderate-to-severe carditis (*vs* none or mild)		12·7 (3·96–40·88)	<0·0001
Age at acute rheumatic fever diagnosis		0·91 (0·82–1·02)	0·10
≥80% adherence to secondary prophylaxis (*vs* <80%)*		1·77 (0·18–17·42)	0·62
Mbarara enrolment site (*vs* Lira)		3·1 (1·1–8·75)	0·033
Mulago enrolment site (*vs* Lira)		2·17 (0·79–5·97)	0·13
≥2 major Jones criteria at time of diagnosis (*vs* <2 criteria)		0·88 (0·35–2·17)	0·78
Jones criteria present at time of diagnosis			
	Monoarthritis	0·4 (0·05–3·00)	0·37
	Polyarthritis	0·67 (0·27–1·70)	0·40
	Polyarthralgia	0·86 (0·39–1·88)	0·70
	Chorea	0·41 (0·05–3·15)	0·39
	ESR elevation	1·47 (0·63–3·42)	0·37
	CRP elevation	2·23 (0·81–6·10)	0·12
	ESR or CRP elevation	1·8 (0·82–3·96)	0·14
	Prolonged PR interval (*vs* normal)	4·37 (1·7–11·23)	0·002
	Anti-streptolysin O antibody elevation	1·28 (0·55–2·99)	0·57
	Anti-DNase B antibody, elevation	0·82 (0·34–1·97)	0·66

CRP=C-reactive protein. ESR=erythrocyte sedimentation rate.

* Model on secondary prophylaxis adherence incorporated only the 67 of 135 patients with complete data and excluded 68 participants with incomplete data.

For the echocardiographic analysis, we excluded 25 (16%) patients who died and therefore could not provide updated data, as well as six (4%) patients whose files were corrupted, leaving 125 (80%) of 156 patients included in this analysis. The rate of any mitral regurgitation was higher at baseline (59 [47%] of 125 patients) than follow-up (52 [42%] of 125 patients). Mitral stenosis of any degree was rare. The rate of any aortic regurgitation was higher at baseline (34 [27%] of 125 patients) than follow-up (19 [15%] of 125 patients). When assigned into categories, at baseline, 78 (63%) of 125 patients had no carditis, 14 (11%) had mild carditis, and 33 (26%) had moderate-to-severe carditis. At follow-up, 93 (74%) of 125 patients had no carditis, 16 (13%) had mild carditis, and 16 (13%) had moderate-to-severe carditis. Therefore, 32 (26%) patients had rheumatic heart disease at follow-up, as evidenced by the presence of any carditis (table 3).

**Table 3:** Baseline and follow-up echocardiogram data for patients previously diagnosed with acute rheumatic fever

	**Patients (n=125)**
**Mitral regurgitation at baseline**	
None	66 (53%)
Trivial	15 (12%)
Mild	15 (12%)
Moderate	12 (10%)
Severe	17 (14%)
**Mitral stenosis at baseline**	
None	124 (99%)
Mild	1 (<1%)
Moderate	0
Severe	0
**Aortic regurgitation at baseline**	
None	91 (73%)
Trivial	5 (4%)
Mild	19 (15%)
Moderate	8 (6%)
Severe	2 (2%)
**Carditis category at baseline**	
None	78 (63%)
Mild	14 (11%)
Moderate-to-severe	33 (26%)
**Mitral regurgitation at follow-up**	
None	73 (58%)
Trivial	23 (18%)
Mild	17 (14%)
Moderate	6 (5%)
Severe	6 (5%)
**Mitral stenosis at follow-up**	
None	122 (98%)
Mild	1 (1%)
Moderate	2 (2%)
Severe	0
**Aortic regurgitation at follow-up**	
None	106 (85%)
Trivial	4 (3%)
Mild	6 (5%)
Moderate	8 (6%)
Severe	1 (1%)
**Carditis category at follow-up**	
None	93 (74%)
Mild	16 (13%)
Moderate-to-severe	16 (13%)

Data are n (%). Patients who died during observation period or who had unavailable follow-up echocardiogram data were excluded.

**Figure 3:**
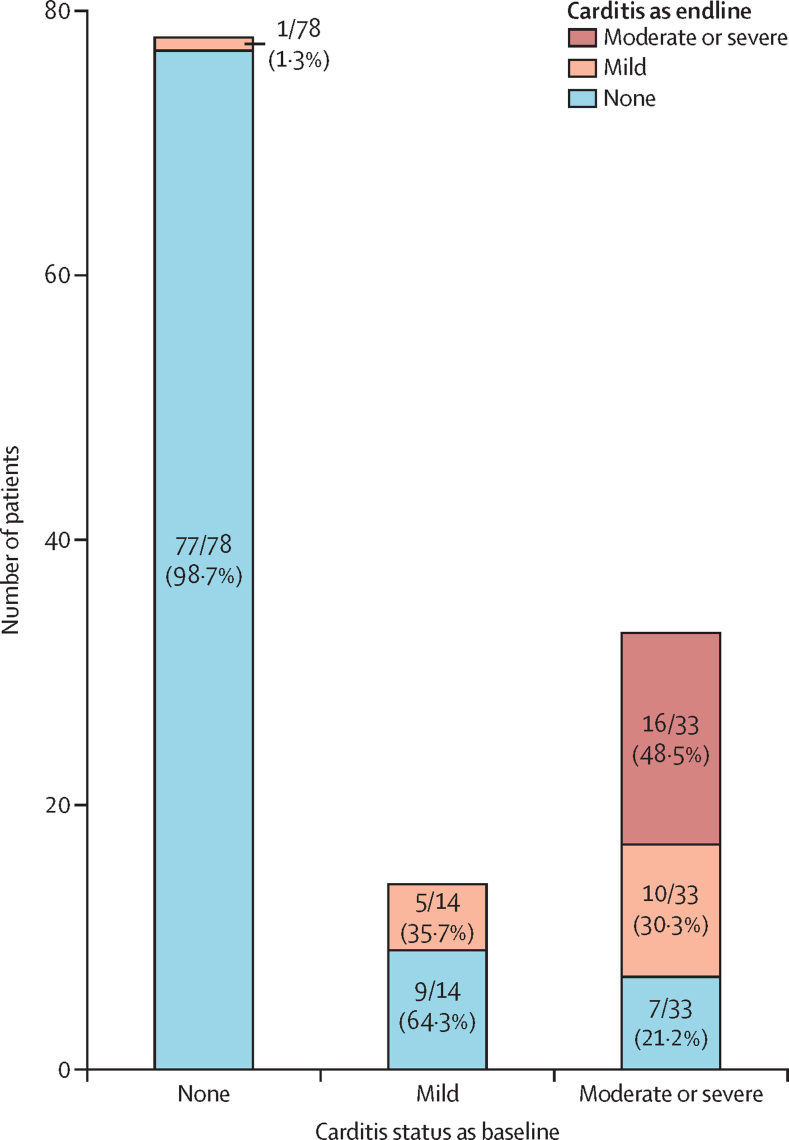
Histogram showing follow-up carditis category as a function of baseline carditis category, according to echocardiogram, in survivors diagnosed with acute rheumatic fever from July 1, 2017, to March 31, 2020, and followed up by the Uganda rheumatic heart disease registry Distribution of carditis category at follow-up for patients who had no carditis, mild carditis, and moderate or severe carditis, respectively, at baseline.

We also assessed follow-up carditis category as a function of the baseline carditis category, where 26 (21%) of 125 patients improved, 93 (74%) remained stable, and one (<1%) progressed (figure 3).

Of the 131 survivors who were included in the secondary antibiotic prophylaxis analysis, all were prescribed intramuscular benzathine penicillin G at the time of diagnosis. However, only 67 (51%) patients had complete data available for review and were included in this subanalysis. Conversely, 64 (49%) patients had incomplete data due to hard-copy records that were lost, damaged, or otherwise incomplete. Of 67 patients with fully available data, 40 (60%) had more than 80% adherence to secondary antibiotic prophylaxis and 27 (40%) had less than 80% adherence to secondary antibiotic prophylaxis. Analysis of the 67 patients with complete data showed no association between adherence category and risk of cardiac-related mortality (HR 2·1 [95% CI 0·2–19·8]).

There was no statistically significant difference between those included in the study and those who were lost to follow-up in regard to age at diagnosis, sex, PR interval, mitral regurgitation, mitral stenosis, aortic regurgitation, or the presence of any major or minor Jones criteria (appendix p 3).

## Discussion

Data in this cohort study provide a negative view of the impact of acute rheumatic fever-related mortality and morbidity in Uganda. Previous data comprise retrospective reviews of only patients who were sick enough to present to a tertiary facility; however, the cohort in this study was recruited from a community setting using active case-finding strategies and so we believe this study provides a uniquely balanced and comprehensive overview of acute rheumatic fever outcomes in sub-Saharan Africa, where its diagnosis remains rare outside of the research context. In this prospective cohort study, over one quarter of the cohort was admitted to hospital and approximately one in six patients succumbed to illness. Many of these deaths occurred within 1–2 years after diagnosis. Only rarely did patients undergo surgery.

Among this cohort, 21 (16%) of 156 patients had a cardiac-related death. This rate was unsurprisingly higher than that reported from rheumatic heart disease-endemic populations within high-income countries, where the most data is available. For example, a retrospective review of hospital records in New Zealand found an overall mortality of 0·68%.^11^ More notably, the mortality rate in our study also exceeded the 4·1% and 12·0% figures reported in studies of patients admitted to hospital from India and Lebanon, respectively, groups which tend to have more severe presentations given their admission to a hospital.^12^, ^13^ When considering incidence, cardiac-related mortality was also quite high at 36·4 per 1000 person-years. Globally, database reviews from Brazil and the Northern Territory of Australia estimated mortality incidences in the first few years after acute rheumatic fever to be 0·27 per 1000 person-years and 0·3 per 1000 person-years, respectively, which are substantially lower than our figure.^14^, ^15^ Similarly, our cohort's incidence of acute rheumatic fever recurrence of 29·4 per 1000 person-years exceeded the 1-year recurrence of 0·03 per 1000 person-years that was separately reported from the Northern Territory of Australia.^16^

We found that death was associated with severe cardiac involvement at initial diagnosis, denoted by PR prolongation on electrocardiogram and moderate-to-severe carditis on echocardiogram, each of which are signs of advanced cardiac disease. We strongly suspect this association is because many patients who were first diagnosed with acute rheumatic fever at the time of study enrolment were actually experiencing only the latest recurrence of multiple previous episodes that had gone undetected and had therefore compounded into rheumatic heart disease. This supposition lends clarity to the early and frequent mortalities in this cohort, which are much more consistent with established outcomes for rheumatic heart disease in Uganda. A recent report investigating 612 Ugandan children with rheumatic heart disease found a 30 month mortality rate that exceeded 25%, an overall mortality rate of 31%, and a median time to death of 7·8 months, figures that are more consistent with our data than those cited for acute rheumatic fever outcomes.^17^

Underdiagnosis of acute rheumatic fever is not specific to Uganda. In fact, in most rheumatic heart disease-endemic regions, acute rheumatic fever is rarely diagnosed and most patients are only identified when they have progressed to rheumatic heart disease with cardiovascular complications. Reasons for underdiagnosis of acute rheumatic fever is multifactorial and includes low clinician and patient awareness and low diagnostic capacity in community settings.^18^, ^19^ A recent study showed that Ugandan clinicians have low health-care readiness to diagnose acute rheumatic fever due to lack of access to imaging and diagnostic tests at the community level and district level.^20^ Our findings suggest that investment in access to echocardiography could improve this readiness and have a great diagnostic benefit, but efforts should not stop there. Other tools are urgently needed. Two newly funded Leducq research networks are focused on developing point-of-care diagnostic tools, such as biomarker assays, that can be deployed at scale to increase the detection rate for acute rheumatic fever within the community setting.

Another likely contributor to the high death rate in our cohort was limited access to surgery, an intervention which improves mortality and morbidity in rheumatic valvulitis. In this cohort, 23 decedents and 22 survivors met expert criteria for valvular surgery at the time of diagnosis because of severe mitral regurgitation or aortic regurgitation.^9^ However, only six patients had surgery. In this region, access to tertiary interventions and specialists is extremely limited, meaning that few patients who warrant advanced care can access it in a timely manner.^18^, ^21^ A recent African Union modelling study highlighted the importance of scaling up secondary and tertiary services for acute rheumatic fever and rheumatic heart disease, including cardiac surgery, with an estimated benefit-to-cost ratio of 4·7 and a net benefit of $US 2·8 billion saved by 2030.^22^ However, scale-up is a complex task, requiring administrative and government support and dedicated human resources, financing, infrastructure, equipment, and pipelines for earlier case detection.^23^ Multisector investment will be required to grow this capacity in the regions where it is needed most.

Our data on echocardiographic outcomes are consistent with the published literature. Generally, carditis in survivors remained stable or improved over time. This is similar to a 5-year prospective study of 35 patients with acute rheumatic fever that reported the presence of mitral regurgitation or aortic regurgitation decrease from 71% to 12%.^24^ A larger study of 103 Turkish children reported improvement or resolution of carditis after 1 year in 66 (64%) children.^25^ Patients with mild disease should expect stability or improvement after acute rheumatic fever. These data also support current American Heart Association/American College of Cardiology guidelines suggesting clinicians should infrequently use surveillance echocardiograms so long as the patient remains asymptomatic.^9^ However, these data do not address the likely substantial long-term risk of rheumatic heart disease in this cohort, which according to one study in Australia was 27% at 1 year, 44% at 5 years, and 52% at 10 years.^16^

Our study has several limitations. First, incomplete data on secondary antibiotic prophylaxis adherence hampered our ability to detect any protective effect of high adherence against negative outcomes, as some studies suggest.^26^, ^27^, ^28^, ^29^ Data that were available showed that half of the patients had low adherence to secondary antibiotic prophylaxis, which is consistent with previous studies in sub-Saharan Africa that attribute suboptimal adherence to barriers that include fear of pain or anaphylaxis, limited health-care access, and low awareness of the detrimental consequences of acute rheumatic fever and rheumatic heart disease.^26^ Second, our study included a relatively small sample size, which led to broad confidence intervals relating to our incidence rates and HRs and hampered our ability to detect risk factors that might have minor, but still important, effects on mortality. Third, our proportion of patients lost to follow-up was larger than anticipated. However, we suspect that their inclusion would not have significantly changed our findings given their similarities in baseline demographic, clinical, and echocardiographic characteristics to the included group. Fourth, there is a possibility of inter-reader variability between initial and follow-up echocardiogram reads, although this was minimised by retaining two of the same three expert readers and reading all echocardiograms in accordance with established guidelines. Finally, because no universal electronic health system exists, most patients hand-carry their records. Therefore, data on interim outcomes were in part collected via verbal interview, which might introduce recall bias to our findings. However, we suspect this effect to be minimal given the significance of these events and our decision to cross-reference these data with the registry.

In summary, our findings provide contemporary community-level data on outcomes of acute rheumatic fever in sub-Saharan Africa. High rates of early mortality from acute rheumatic fever require urgent action to improve prevention, diagnosis, and treatment for this largely preventable childhood illness. Our findings also highlight the feasibility and importance of using decentralised, community-based acute rheumatic fever education and screening programmes to improve diagnosis of acute rheumatic fever, and to capture more patients at the time of initial diagnosis when the avoidance of recurrent acute rheumatic fever might improve outcomes.

## Equitable partnership declaration

## Data sharing

De-identified participant data will be available through reasonable request to the corresponding author.

## Declaration of interests

TP reports grant funding from the Wellcome Fund (grant number 222098/Z/20/Z). All other authors declare no competing interests.

## Supplementary Materials

Supplementary appendix 1

Equitable Partnership Declaration
