# Supplementary material for: Outcomes of rheumatic fever in Uganda: a prospective cohort study
Source: Lancet Glob Health. 2024 Feb 14;12(3):e500–8. doi: 10.1016/S2214-109X(23)00567-3 (PMC10882210; doi:10.1016/S2214-109X(23)00567-3)
Supplement: Supplementary appendix 1 [file mmc1.pdf]

THE LANCET
Global Health

Supplementary appendix 1

This appendix formed part of the original submission and has been peer reviewed. We post it as supplied by the authors.

Supplement to: Wirth SH, Pulle J, Seo J, et al. Outcomes of rheumatic fever in Uganda: a prospective cohort study. *Lancet Glob Health* 2024; **12:** e500–08.

Appendix

Page 2: Appendix 1: Kaplan-Meier survival curve showing freedom from cardiac-related mortality for Ugandan patients aged 4-23 years who were diagnosed with acute rheumatic fever from July 1, 2017 to March 31, 2020 and followed by the Uganda rheumatic heart disease registry

Page 3: Appendix 2: Baseline characteristics of included and excluded Ugandan patients aged 4-23 years who were diagnosed with acute rheumatic fever from July 1, 2017 to March 31, 2020, demonstrating distribution of demographic variables, PR interval, echocardiographic variables, and major and minor Jones criteria at the time of acute rheumatic fever diagnosis.

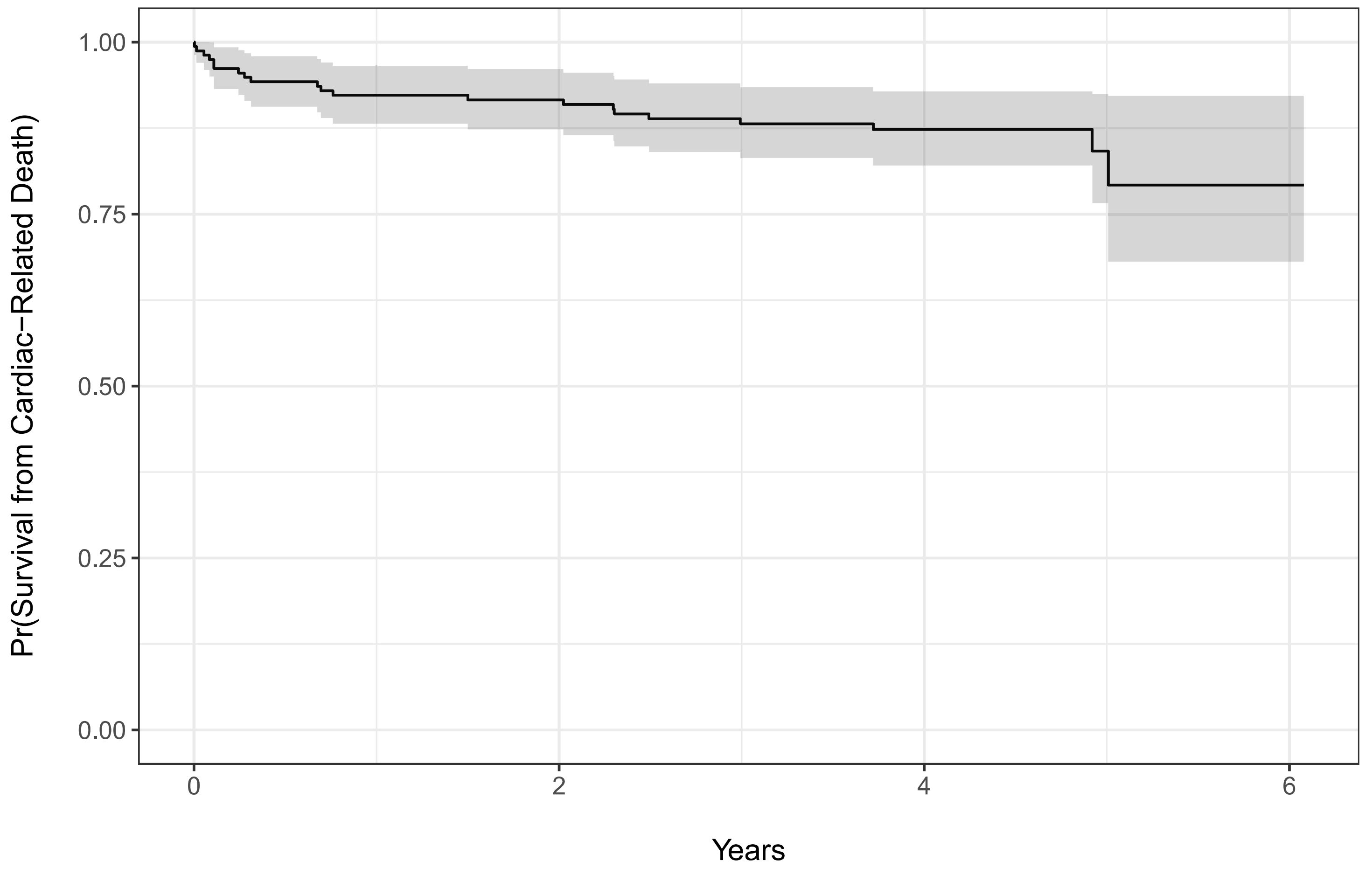

At Risk	156	136	90	2
Events	0	13	19	21

Characteristic	Excluded (n=26)	Included (n=156)	p
Age at initial diagnosis (years), mean (SD)	9.65 (3.6)	9.62 (3.7)	0.96
Female sex, n (%)	14 (55)	76 (49)	0.47
Enrollment site, n (%)			**0.0017**
Lira	9 (35)	105 (67)	
Mulago	12 (46)	26 (17)	
Mbarara	5 (19)	25 (16)	
PR interval category, n (%)			0.47
Normal	25 (96)	140 (90)	
Prolonged	1 (4)	16 (10)	
Mitral regurgitation, n (%)			0.28
None	16 (62)	72 (46)	
Trivial	5 (19)	17 (11)	
Mild	1 (4)	15 (10)	
Moderate	1 (4)	14 (9)	
Severe	3 (12)	38 (24)	
Mitral stenosis, n (%)			1.0
None	26 (100)	150 (96)	
Mild	0	2 (1)	
Moderate	0	1 (1)	
Severe	0	3 (2)	
Aortic regurgitation, n (%)			0.45
None	21 (81)	105 (67)	
Trivial	2 (7)	7 (5)	
Mild	3 (12)	22 (14)	
Moderate	0	14 (9)	
Severe	0	7 (5)	
Number of Major Jones Criteria, n (%)			0.15
One	23 (80)	108 (69)	
Two	3 (12)	41 (26)	
Three	0	7 (5)	
Positive Major Jones Criteria			
Monoarthritis, n (%)	1 (4)	12 (8)	0.69
Polyarthritis, n (%)	4 (15)	49 (31)	0.11
Polyarthralgia, n (%)	19 (73)	81 (52)	0.055
Subcutaneous nodules, n (%)	0	1 (1)	1.0
Erythema marginatum, n (%)	0	0	n/a
Chorea, n (%)	1 (4)	11 (7)	1.0
Positive Minor Jones Criteria	25 (96)	146 (94)	1.0
Fever, n (%)			
Monoarthralgia, n (%)	0	2 (1)	1.0
ESR elevation, n (%)	17 (65)	94 (60)	0.67
CRP elevation, n (%)	19 (73)	97 (62)	0.38
Elevated or rising ASO titers, n (%)	13 (50)	96 (62)	0.29
Elevated or rising ADB titers, n (%)	22 (65)	112 (72)	0.23

Notes: p-values obtained from independent samples t-tests or Fisher's exact tests.
