# Supplementary material for: Outcomes of rheumatic fever in Uganda: a prospective cohort study
Source: Lancet Glob Health. 2024 Feb 14;12(3):e500–8. doi: 10.1016/S2214-109X(23)00567-3 (PMC10882210; doi:10.1016/S2214-109X(23)00567-3)
Supplement: Equitable Partnership Declaration [file mmc2.pdf]

THE LANCET
Global Health

Supplementary appendix 2

This Equitable Partnership Declaration (EPD) was submitted by the authors, and we reproduce it as supplied. It has not been peer reviewed. *The Lancet*'s editorial processes have not been applied to the EPD.

Supplement to: Wirth SH, Pulle J, Seo J, et al. Outcomes of rheumatic fever in Uganda: a prospective cohort study. *Lancet Glob Health* 2024; **12:** e500–08.

Equitable Partnership Declaration questions

Researcher considerations

1. Please detail the involvement that researchers who are based in the region(s) of study had during a) study design; b) clinical study processes, such as processing blood samples, prescribing medication, or patient recruitment; c) data interpretation; and d) manuscript preparation, commenting on all aspects. If they were not involved in any of these aspects, please explain why.

   This question is intended for international partnerships; if all your authors are based in the area of study, this question is not applicable.

   This should include a thorough description of their leadership role(s) in the study. Are local researchers named in the author list or the acknowledgements, or are they not mentioned at all (and, if not, why)? Please also describe the involvement of early career researchers based in the location of the study. Some of this information might be repeated from the Contributors section in the manuscript. Note: we adhere to [ICMJE authorship criteria] when deciding who should be named on a paper.

**a) Study design:**<br>This study was primarily conceived by Dr. Ndagire who is the senior author of the project and was a co-author and lead investigator on the two preceding parent studies to this project. Study design was shared between Drs. Wirth, Beaton, Ndagire, Okello, and Mr. Pulle. Drs. Ndagire and Okello work at the Uganda Heart Institute in Kampala, Uganda as clinical cardiologists. Mr. Pulle works at the Uganda Heart Institute as a research coordinator and PhD candidate. Dr. Wirth and Dr. Beaton work at Cincinnati Children's Hospital as a clinical fellow and professor of pediatrics, respectively. All parties, as well as several other authors on this manuscript, are members and teammates within the Rheumatic Heart Disease Research Collaborative in Uganda (RRCU, rrcuganda.org). This collaborative is comprised primarily of North America-based and Uganda-based clinical researchers with shared vision of reducing the global burden of RHD through research and implementation projects performed in Uganda.
**b) Clinical study processes:**<br><br>Dr. Ndagire and Mr. Pulle were primarily responsible for organizing re-recruitment of participants to return for the August 2022 visit, both by personally calling many participants and otherwise directing a team of research support staff on the ground in Uganda to do so. After recruitment, participants returned for a dedicated visit follow-up where they underwent a verbal interview, EKG, echo, and registry review. Echos were later read by an adjudication panel. These verbal interviews were conducted in English and the local languages by Dr. Ndagire, Mr. Pulle, and Dr. Wirth on site at the three enrolment sites, as well as the aforementioned team of Ugandan research support staff. EKGs were performed by Mr. Pulle and the support staff. Echocardiograms were performed by Dr. Ndagire and Dr. Wirth. Echo readers/adjudication occurred by Dr. Ndagire, Dr. Nakagaayi (a junior cardiologist/clinician at the Uganda Heart Institute and team member with the RRCU), and Dr. Sable (co-founder and senior consultant to the RRCU who has been performing research in Uganda in partnership with local collaborators for over 20 years). Finally, Dr. Wirth, Dr. Ndagire, and Mr. Pulle jointly conducted registry review.

**c) Data interpretation:**<br>Drs. Wirth, Beaton, Ndagire, and Olberding and Mr. Jafesi Pulle all contributed to data interpretation. The majority of data interpretation was led by Drs. Wirth, Beaton, Ndagire, and Pulle. Dr. Ndagire provided clinical insight in how to interpret the data for the secondary outcome measures of echocardiographic change over time and secondary antibiotic prophylaxis adherence, based upon her clinical experience following these children. Mr. Pulle provided leadership in assessing the mortality data, as in his role and primary research coordinator in the RRCU and its overlap into the RHD Registry, he is a primary point of contact for many of the patients in the registry.
**d) Manuscript preparation:**<br>Drs. Wirth, Beaton, and Ndagire were primarily responsible for initial manuscript preparation. Dr. Nakagaayi, Dr. Okello, and Mr. Pulle provided critical review and workshopped specific sections regarding echocardiographic data and clinical processes given their heavy involvement with those portions of the study.

2. Were the data used in your study collected by authors named on the paper, or have they been extracted from a source such as a national survey? ie, is this a secondary analysis of data that were not collected by the authors of this paper. If the authors of this paper were not involved in data collection, how were data interpreted with sufficient contextual knowledge?

   The Lancet Global Health *believe contextual understanding is crucial for informed data analysis and interpretation.*

Data from this study comprised two sources. The first was primary data collected in a prospective fashion at the time of study follow-up visit by Dr. Wirth, Dr. Ndagire, and Mr. Pulle. The second was secondary analysis of data collected from the Uganda RHD Registry at the time of the initial ARF diagnosis (start of the prospective observational period for this study). The RHD Registry is a recent initiative (within the past five years) that started as a research database constructed and maintained jointly by the investigators within the RRCU and recently has transitioned to becoming the primary clinical registry of Ugandan patients with ARF or RHD and now is jointly sponsored by the Ugandan Ministry of Health as those patients' electronic health record. All secondary data from the registry, therefore, is compiled by Ugandan clinicians and investigators. In this project specifically, all data we used in secondary analysis was first entered during the time of the two parents studies to this project under the supervision of the same investigators who are co-authors on this project. Given that our study authors (Dr. Ndagire, Dr. Okello, and Mr. Pulle) were responsible for collecting this data, and because they continue to intimately interact with this data in both the research and clinical context, we believe there is appropriate local, contextual knowledge to interpret these data correctly.

3. How was funding used to remunerate and enhance the skills of researchers and institutions based in the area(s) of study? And how was funding used to improve research infrastructure in the area of study?

   Potentially effective investments into long-term skills and opportunities within institutions could include training or mentorship in analytical techniques and manuscript writing, opportunities to lead all or specific aspects of the study, financial remuneration rather than requiring volunteers, and other professional development and educational opportunities.

Improvements to research infrastructure could be funding of extended trial designs (such as platform trials) and use of master protocols to enable these designs, establishment of long-term contracts for research staff, building research facilities, and local control of funding allocation.

**Skills:** There were several sources of funding for this project. Primary support was given through the Arnold W. Strauss award at Cincinnati Children's Hospital. This is an intramural grant through the department of pediatrics awarded to Dr. Wirth under Dr. Beaton's mentorship. However, given that Dr. Wirth and Dr. Beaton are team members in the RRCU, all funds not reserved for travel and statistical support was turned over the RRCU business team (i.e., given over for local control of funding allocation). Those funds were distributed as follows- financial renumeration of participants, salary support for dedicated research staff (research RN's and assistants who directly participated in the recruitment and research processes under this project), and certain research equipment such as EKG machine paper, echo device gel, and the printing of hard-copy research documentation, consents, and data collection instruments.<br><br>Additional funding support was provided by the Leducq ARC Network and the AHA SFRN grants. These funds were more generally directed toward supporting the skills of the RRCU research team. These include training and mentorship in conducting prospective trials, manuscript writing, the support of long-term contracts for research staff, and establishing/staffing research clnics around Uganda.
**Research infrastructure:** There were several sources of funding for this project. Primary support was given through the Arnold W. Strauss award at Cincinnati Children's Hospital. This is an intramural grant through the department of pediatrics awarded to Dr. Wirth under Dr. Beaton's mentorship. However, given that Dr. Wirth and Dr. Beaton are team members in the RRCU, all funds not reserved for travel and statistical support was turned over the RRCU business team (i.e., given over for local control of funding allocation). Those funds were distributed as follows- financial renumeration of participants, salary support for dedicated research staff (research RN's and assistants who directly participated in the recruitment and research processes under this project), and certain research equipment such as EKG machine paper, echo device gel, and the printing of hard-copy research documentation, consents, and data collection instruments.<br><br>Additional funding support was provided by the Leducq ARC Network and the AHA SFRN grants. These funds were more generally directed toward supporting the skills of the RRCU research team. These include training and mentorship in conducting prospective trials, manuscript writing, the support of long-term contracts for research staff, and establishing/staffing research clnics around Uganda.

4. How did you safeguard the researchers who implemented the study?

Please describe how you guaranteed safe working conditions for study staff, including provision of appropriate personal protective equipment, protection from violence, and prevention of overworking.

Follow-up visits (i.e. all direct participant interaction) occurred at one of three existing clinical sites, all being regional referral hospitals in either Mulago, Lira, or Mbarara. Therefore, PPE was provided through the auspices of these existing infrastructure of these regional referral hospitals. This equipment included masks, gloves, and gowns.

Similarly, existing third-party security, already in place and employed by the regional referral hospitals, was utilized for protection of study personnel.

Overworking was addressed through appropriate scheduling. Participants were recruited and asked to return for a dedicated follow-up visit that fell within a pre-determined schedule over a period of three consecutive weeks. Only 135 participants returned for dedicated follow-up, equating to an average of 9 participants seen per study day. Each study visit required ~30 minutes to perform procedures, equating to an average of 4.5 hours per day to conduct study processes. During these scheduled days, study personnel were not responsible for non-research tasks, thereby freeing them to focus only on study procedures. On all days, study personnel were provided with a break for breakfast and lunch each day, and lunch was purchased for each study team member through the available funded monies.

Benefits to the communities and regions of study

5. How does the study address the research and policy priorities of its location?

How were the local priorities determined and then used to inform the research question? Who decided which priorities to take forward? Which elements of the study address those priorities?

Rheumatic heart disease is a major contributor to acquired cardiovascular mortality and disability adjusted life years in Uganda, just as it is globally. About 1% of Ugandans have RHD (likely an underestimate). Recently, the Ugandan Ministry of Health, in response to several international resolutions such as the 2018 WHO Resolution on RHD, has prioritized the need to improve the knowledge of and care for ARF and RHD in its country. In doing so, it has partnered with the Uganda Heart Institute and the RRCU to conduct several epidemiological studies and interventional trials. This project was borne of that partnership and through key collaborators in that partnership, including Dr. Beaton, Dr. Okello, Dr. Ndagire, and Mr. Pulle, was designed so as to meet relevant aims that of value within the local context.

6. How will research products be shared in the community of study?

For instance, will you be providing written or oral layperson summaries for non-academic information sharing? Will study data be made available to institutions in the region(s) of study? The Lancet Global Health *encourages authors to translate the summary (abstract) into relevant languages after paper editing; do you intend to translate your summary?*

Because this study is a product of the RRCU, which is a collaborative of co-equal North American and local Ugandan researchers, the RRCU. Therefore the Uganda Heart Institute and its clinicians will have unfettered access to this manuscript and the data contained there within. has co-equal ownership over this manuscript and the data contained there within. Oral and written layperson summaries will be made available for information sharing, particularly for patients of the Uganda Heart Institute and to relevant future research populations in upcoming RRCU studies/trials.

Uganda is a country with over 40 different native languages. The common language for medicine and research is English. Therefore, the summary will be maintained in English for dissemination among our local collaborators in the RRCU and Uganda Heart Institute. As referenced above, oral and lay summaries will be produced for dissemination among the local population when being used for education and awareness raising in future clinical or research contexts

7. How were individuals, communities, and environments protected from harm?
Because this was not an interventional study but rather an observational study, there was minimal risk involved in participation. However, appropriate protocols for the conduction of ethical research practices were followed. We obtained ethical approval from the Uganda Heart Institute ethical review board, each participant and/or his or her caregivers were re-consented or re-assented for this study, there was no cost to participate in this study (and in fact participants were reimbursed for their time and travel), and participants actually could have received some benefit from the study in that they received updated clinical review and echocardiography to survey the status of their heart disease. Those that were found to have concerning findings were re-referred to clinical care.

a) How did you ensure that sensitive patient data was handled safely and respectfully? Was there any potential for stigma or discrimination against participants arising from any of the procedures or outcomes of the study?

All data was handled in a strictly confidential manner by the research team. All participant records were de-identified to designated ID numbers. Initial hard copy data collection forms were entered into a secure, password protected, online REDCap database and then original hard copy forms were stored in a locked file cabinet within the offices of the RRCU and these were made available at request for auditing. No information was released to any unauthorized third party.

This was an observational study, the protocol for which comprised a one-day study visit where participants underwent a verbal history, electrocardiogram, echocardiogram, and review of hard copy secondary antibiotic prophylaxis records. All participants were submitted to the same protocol without variation. Each participant's study visit occurred in privacy behind a locked door, and there was no potential for inappropriate sharing of study data to other participants or other

third parties. Therefore, there was no potential for stigma or discrimination arising from the data that was collected.

b) *Might any of the tests be experienced as invasive or culturally insensitive?*

No tests were invasive. The study procedures were developed in collaboration with the Ugandan members of the RRCU and therefore were vetted for cultural insensitivity.

c) *How did you determine that work was sensitive to traditions, restrictions, and considerations of all cultural and religious groups in the study population?*

This was an observational study and so no interventions were delivered that might stigmatize any participants. All participants in this study already carried the diagnosis of ARF and were already participating in routine clinical follow-up, so the study did not introduce anything specifically new to these participants that they had not already experienced and consented to. Nonetheless, all study participants were subjected to an updated consent and/or assent process in the language of their preference, allowing for potential participants to ask clarifying questions and raise objections to participation. Finally, all study procedures were developed in collaboration with the co-equal Ugandan members of the RRCU and therefore were vetted for cultural insensitivity from members of the Uganda culture.

d) *Were biowaste and radioactive waste disposed of in accordance with local laws?*

There was no biowaste or radioactive waste. ECG and echocardiography machines were cleaned between each participant using sanitizing solutions and wiped off using tissue paper, which was disposed of in refuse containers in accordance with local laws.

e) *Were any structures built that would have impacted members of the community or the environment (such as handwashing facilities in a public space)? If so, how did you ensure that you had appropriate community buy-in?*

None. All study procedures were conducted at official health clinics or regional referral hospitals that were already in place.

f) *How might the study have impacted existing health-care resources (such as staff workloads, use of equipment that is typically employed elsewhere, or reallocation of public funds)?*

The study procedures were conducted in their entirety by the RRCU study team, which was comprised of the lead investigators (Dr. Wirth, Dr. Ndagire, Mr. Pulle) and local, on-site study staff (research nurses). Therefore, no time was taken away from the clinical staff working at those locations. We provided our own ECG and echocardiography machines to ensure that equipment for our study was not taken from the general clinical equipment. The funding was provided in entirety by grant funding rather than public funds.

8. Finally, please provide the title (eg, Dr/Prof, Mr/Mrs/Ms/Mx), name, and email address of an author who can be contacted about this statement. This can be the corresponding author.

Name: Dr. Scott H. Wirth, MD
Email: scott.wirth@cchmc.org
